# The neuroscience basis and educational interventions of mathematical cognitive impairment and anxiety: a systematic literature review

**DOI:** 10.3389/fpsyg.2023.1282957

**Published:** 2023-11-30

**Authors:** Hao Yu

**Affiliations:** Faculty of Education, Shaanxi Normal University, Xi'an, Shaanxi, China

**Keywords:** mathematical cognitive impairment, mathematical anxiety, neuroscience, educational interventions, systematic literature review

## Abstract

**Introduction:**

Mathematics is a fundamental subject with significant implications in education and neuroscience. Understanding the cognitive processes underlying mathematical cognition is crucial for enhancing educational practices. However, mathematical cognitive impairment and anxiety significantly hinder learning and application in this field. This systematic literature review aims to investigate the neuroscience basis and effective educational interventions for these challenges.

**Methods:**

The review involved a comprehensive screening of 62 research articles that meet the ESSA evidence levels from multiple databases. The selection criteria focused on studies employing various methodologies, including behavioral experiments and neuroimaging techniques, to explore the neuroscience underpinnings and educational interventions related to mathematical cognitive impairment and anxiety.

**Results:**

The review identified key themes and insights into the neuroscience basis of mathematical cognitive impairment and anxiety. It also examined their impact on educational practices, highlighting the interplay between cognitive processes and educational outcomes. The analysis of these studies revealed significant findings on how these impairments and anxieties manifest and can be addressed in educational settings.

**Discussion:**

The review critically analyzes the shortcomings of existing research, noting gaps and limitations in current understanding and methodologies. It emphasizes the need for more comprehensive and diverse studies to better understand these phenomena. The discussion also suggests new directions and potential improvement strategies for future research, aiming to contribute to more effective educational interventions and enhanced learning experiences in mathematics.

**Conclusion:**

This systematic review provides valuable insights into the neuroscience basis of mathematical cognitive impairment and anxiety, offering a foundation for developing more effective educational strategies. It underscores the importance of continued research in this area to improve educational outcomes and support learners facing these challenges.

## 1 Introduction

Mathematics, as a universal and foundational subject, has extensive applications in various fields such as society, technology, and economics. Particularly in education and neuroscience, the study of mathematical cognition has gradually become an important research object (Sotiropoulos, 2014). This involves complex issues of how humans acquire, process, and apply mathematical knowledge (Moustafa et al., 2017). In education, exploring the processes and mechanisms of mathematical cognition helps optimize teaching methods, improve students’ learning processes, and enhance overall teaching effectiveness (Gilmore, 2023; Medrano and Prather, 2023). Simultaneously, in neuroscience, studying the neural basis of mathematical cognition allows us to understand the operating mechanisms of the brain more deeply (Matejko and Ansari, 2015; Looi et al., 2016). However, in the research and application of mathematical cognition, mathematical cognitive impairment and mathematical anxiety are two universal and severe problems (Moustafa et al., 2019). Mathematical cognitive impairment often leads to persistent difficulties in mathematical learning and application, severely affecting academic achievements and potentially negatively impacting future career development and social adaptability. Mathematical anxiety typically manifests as tension and anxiety when individuals face mathematical tasks, likely exacerbating the problems of mathematical cognitive impairment (Henschel and Roick, 2020). To explore the causes, manifestations, and intervention methods of these two problems, researchers have conducted extensive research using various methods such as behavioral experiments and neuroimaging, achieving some research results. However, there are still many unresolved issues and deficiencies regarding the neuroscience basis of mathematical cognitive impairment and anxiety and how to effectively alleviate these two problems through educational interventions.

This paper aims to explore and analyze the neuroscience basis of mathematical cognitive impairment and anxiety and how these bases affect the methods and effects of educational practices by reviewing related literature. It will focus on exploring the neural level performance characteristics of mathematical cognitive impairment and anxiety, their mutual influences, and effective ways to alleviate these problems through educational interventions. It will also summarize and analyze the shortcomings of existing research, aiming to find new directions and possible improvement strategies for future research. We hope this paper can provide valuable references and inspirations for the theory and practice of mathematical education.

## 2 Methods

**Figure 1:**
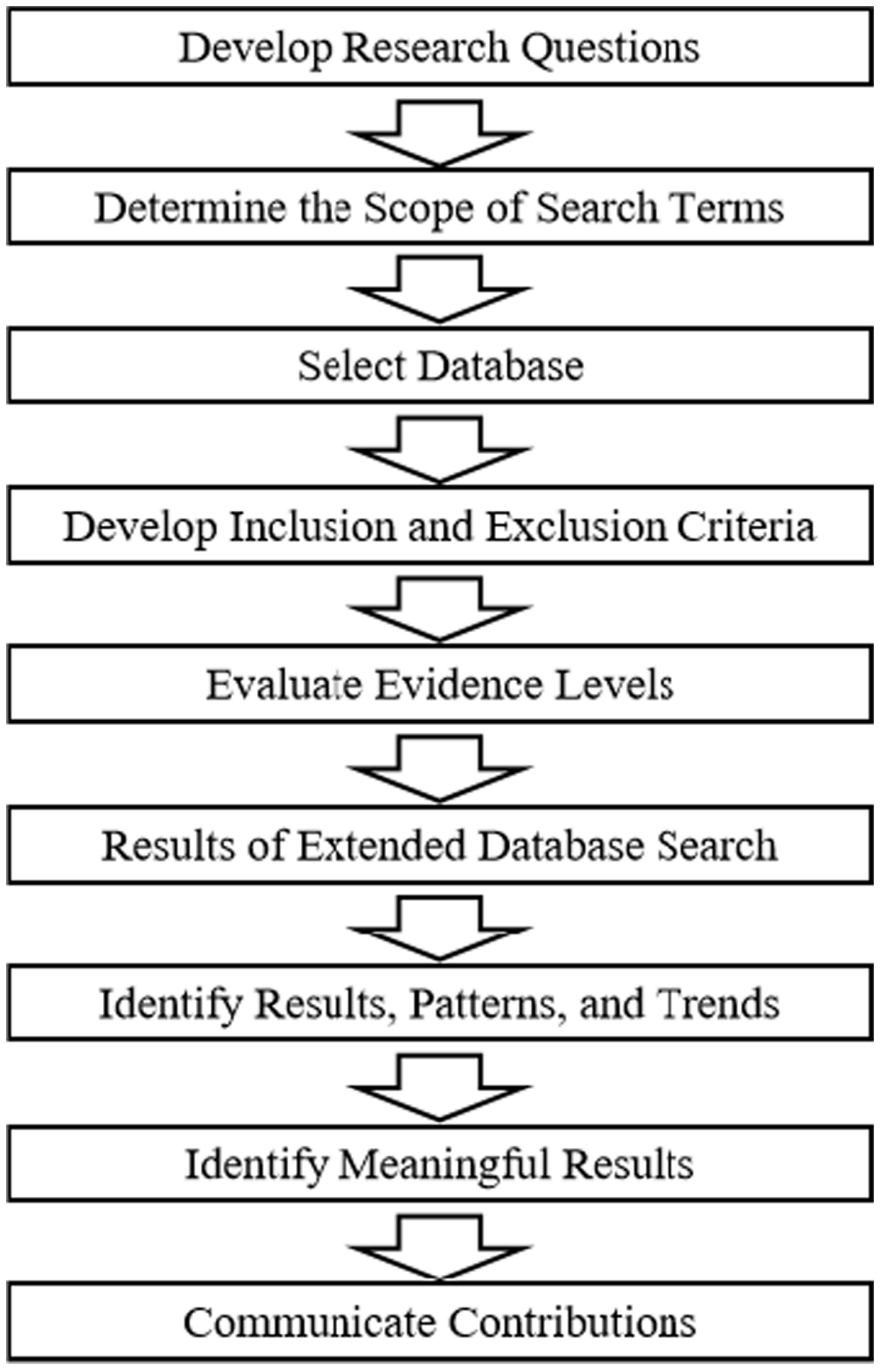
Systematic literature review process.

The objective of this systematic literature review is to construct an understanding of mathematical anxiety and cognitive impairment from the existing research foundation, aiming to identify significant themes within the current knowledge base. Our review process included formulating research questions; determining the scope of search terms; selecting databases; establishing reasonable inclusion and exclusion criteria; evaluating evidence levels; expanding database search results; identifying meaningful outcomes, patterns, and trends; and conveying contributions(see Figure 1).

For this study, we utilized several search engines: Web of Science, EBSCO, Scopus, and ERIC. These engines were chosen based on their breadth and tendency to be more comprehensive than other databases. The initial primary search terms used were “mathematics,” “math education,” “mathematical,” and “math cognition,” each paired with “neuroscience,” “anxiety,” and “cognitive impairment.” We did not use a specific year range for the search, opting instead for an open search to observe a more comprehensive representation. The preliminary search yielded 2,328 articles. From the results, 419 duplicate articles were removed. Our systematic literature review included a snowball sampling strategy, identifying additional studies by reviewing citations from articles in our preliminary search, incorporating an additional 29 articles, totaling 1938. The screening process involved two rounds of review, applying our search inclusion and exclusion criteria to narrow down the pool of articles suitable for inclusion. The first round of screening primarily focused on the titles and abstracts of the articles, excluding 1,526 articles, leaving 369. Table 1 provides examples of the excluded articles.

**Table 1:** Sample of excluded articles.

Sample of excluded articles	
*Non mathematical neuroscience*	
**1**	Abbott, L. F. (2008). Theoretical neuroscience rising. Neuron, 60(3), 489–495.
**2**	Silva, A. C., Tomassini, C., Zurbrigg, J., Palacios, A., Amarante, V., & Bouzat, C. (2021). Gender inequality in Latin American Neuroscience community. IBRO Neuroscience Reports, 10, 104–108.
**3**	Sizemore, A. E., Phillips-Cremins, J. E., Ghrist, R., & Bassett, D. S. (2019). The importance of the whole: Topological data analysis for the network neuroscientist. Network Neuroscience, 3(3), 656–673.
**4**	Baker, D. P., Salinas, D., & Eslinger, P. J. (2012). An envisioned bridge: Schooling as a neurocognitive developmental institution. Developmental Cognitive Neuroscience, 2, S6–S17.
**5**	Silver, R., Boahen, K., Grillner, S., Kopell, N., & Olsen, K. L. (2007). Neurotech for neuroscience: unifying concepts, organizing principles, and emerging tools. The Journal of Neuroscience, 27(44), 11,807–11,819.
**…**	
*Not paying attention to mathematics education*	
**1**	McCollum, G. (2003). Mathematics reflecting sensorimotor organization. Biological Cybernetics, 88(2), 108–128.
**2**	Gutkin, B., Pinto, D., & Ermentrout, B. (2003). Mathematical neuroscience: from neurons to circuits to systems. Journal of Physiology-paris, 97(2–3), 209–219.
**3**	Amigó, J. M., & Small, M. (2017). Mathematical methods in medicine: neuroscience, cardiology and pathology. Philosophical Transactions of the Royal Society A, 375(2096), 20,170,016.
**4**	Tallant, J. (2013). Pretense, Mathematics, and Cognitive Neuroscience. The British Journal for the Philosophy of Science, 64(4), 817–835.
**5**	Feng, S. F., & Holmes, P. (2016). Will big data yield new mathematics? An evolving synergy with neuroscience. Ima Journal of Applied Mathematics, 81(3), 432–456.
**…**	
*Not paying attention to mathematical cognitive impairment and mathematical anxiety*	
**1**	Van Nes, F. (2011). Mathematics Education and Neurosciences: Towards interdisciplinary insights into the development of young children’s mathematical abilities. Educational Philosophy and Theory, 43(1), 75–80.
**2**	Ng, S. S. N., & Rao, N. (2010). Chinese number words, culture, and mathematics learning. Review of Educational Research, 80(2), 180–206.
**3**	Anderson, O. R., Love, B. C., & Tsai, M. J. (2014). Neuroscience Perspectives for science and Mathematics Learning in Technology-Enhanced Learning Environments. International Journal of Science and Mathematics Education, 12(3), 467–474.
**4**	Cuturi, L. F., Cappagli, G., Yiannoutsou, N., Price, S., & Gori, M. (2021). Informing the design of a multisensory learning environment for elementary mathematics learning. Journal on Multimodal User Interfaces, 16(2), 155–171.
**5**	Wilkey, E. D., Cutting, L. E., & Price, G. R. (2017). Neuroanatomical correlates of performance in a state-wide test of math achievement. Developmental Science, 21(2).
**…**	

**Figure 2:**
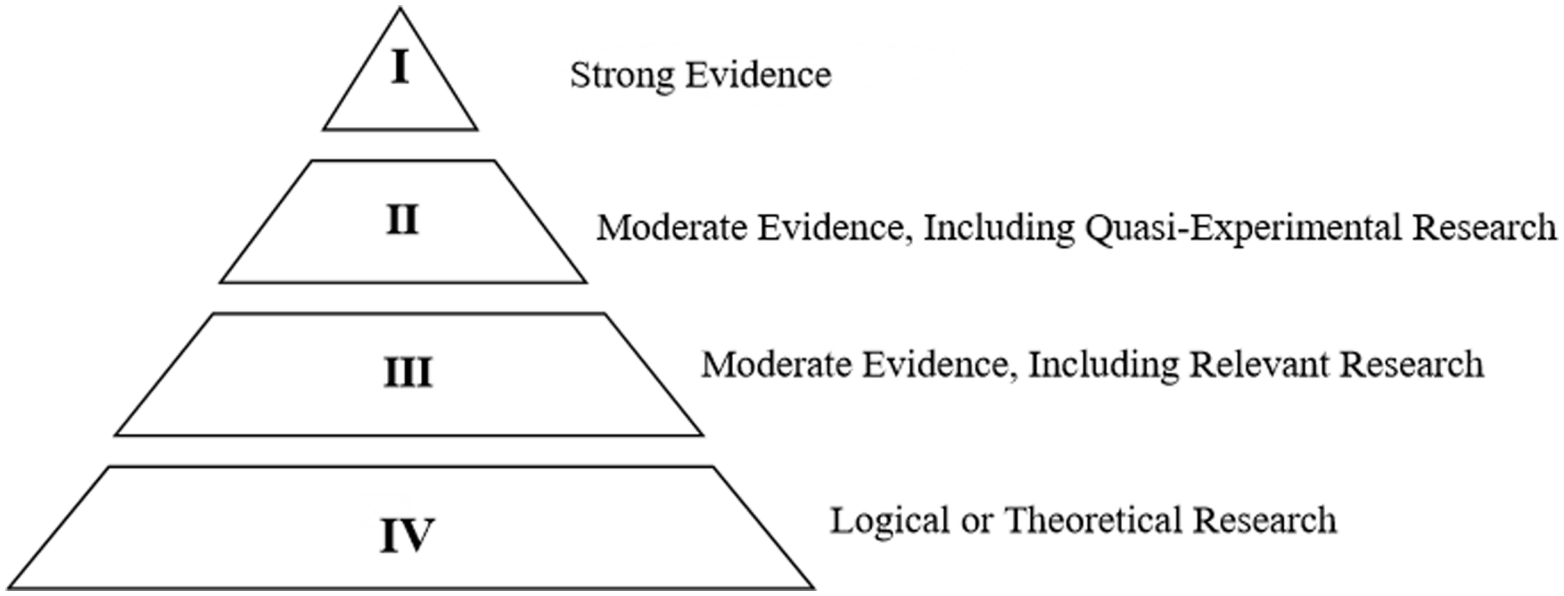
ESSA Evidence Hierarchy.

The second round of screening involved using the ESSA evidence levels to assess the eligibility of full-text articles, determining which articles should be included in the review based on the rigor of the research (see Figure 2) (Thomas and Harden, 2008). 307 articles were excluded as they did not meet the ESSA evidence levels. The final 62 articles included: 7 meeting ESSA Tier 1, 9 meeting ESSA Tier 2, 15 meeting ESSA Tier 3, and 31 meeting ESSA Tier 4.

**Figure 3:**
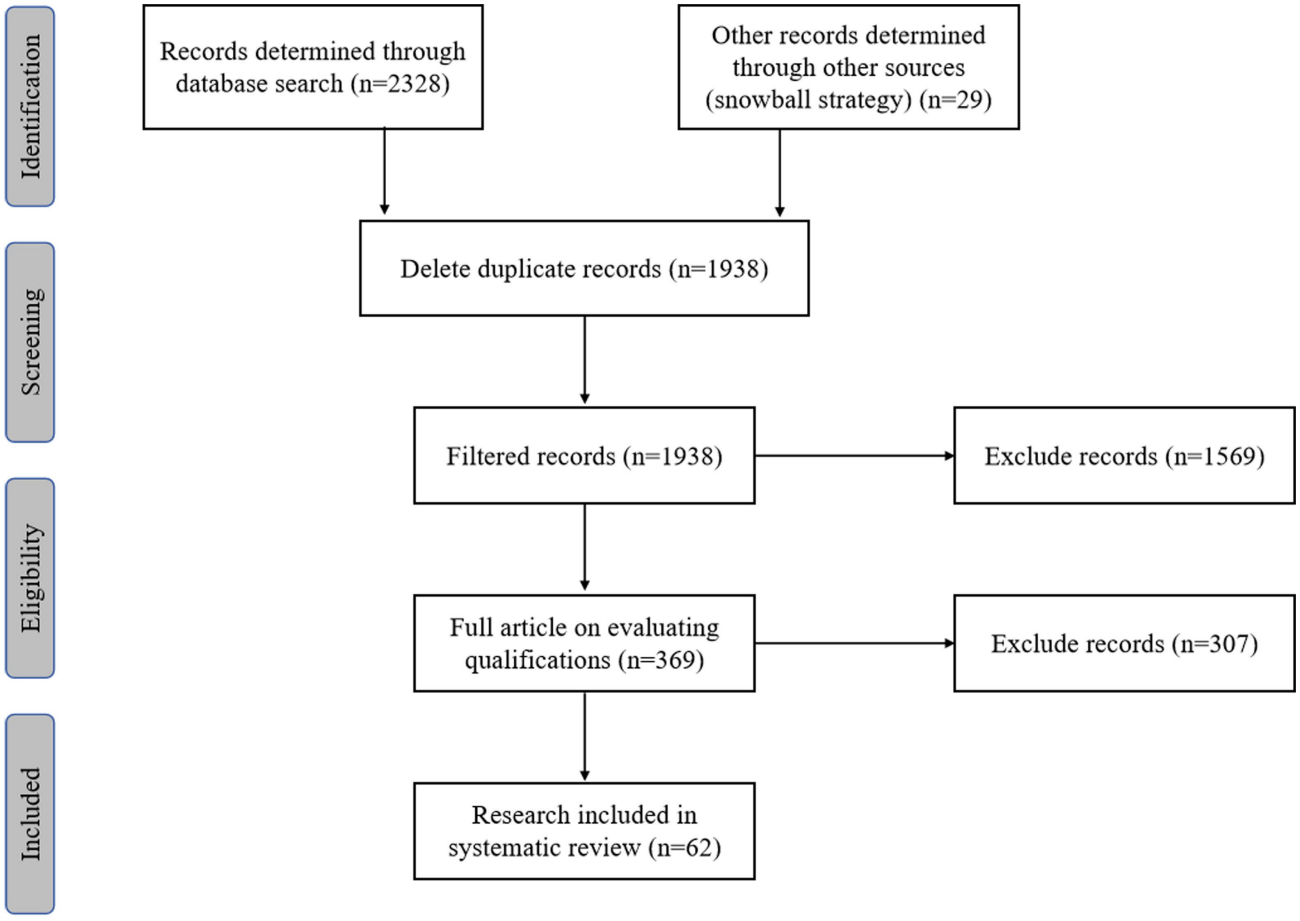
Preferred reporting items for PRISMA diagram of selection process.

We employed the Preferred Reporting Items for Systematic Reviews and Meta-Analyses (PRISMA) framework, an evidence-based standard for systematic reviews and meta-analyses, to guide the selection and analysis of literature methods. Figure 3 displays a PRISMA flow diagram summarizing the process used to identify studies.An information database was constructed using the articles included in the systematic literature review. The database comprised general information (e.g., article titles, authors, publication dates, publications, and abstracts), research types (e.g., research questions, methods), and summaries of findings. The authors used the PRISMA guidelines to elucidate the data collection and analysis methods for each study.

We did not use a conceptual framework to construct the coding process but employed thematic synthesis to analyze the articles, where themes were generated from the primary studies. The three stages of this method were: (1) coding selected texts, (2) developing descriptive themes, and (3) generating analytical themes. For each article, findings, discussions, and implications were independently coded by the authors of this manuscript and then discussed collectively. Emergent codes were identified, descriptive themes developed, and analytical themes generated. After a rigorous screening process and analysis, we selected a series of primary literatures on mathematical anxiety and cognitive impairment. These literatures mainly fall into several categories (see Table 2).

**Table 2:** Main classification of literature.

Theme	Content	Number of documents	Quantity proportion
Relationship Between Mathematical Anxiety and Cognitive Function	Focuses on how mathematical anxiety impacts individual cognitive functions such as working memory, attention, and executive functions.	37	59.7%
Assessment and Intervention for Mathematical Anxiety	Investigates the assessment and intervention strategies for mathematical anxiety, including various assessment tools and intervention approaches.	25	40.3%
Diagnosis and Characteristics of Mathematical Learning Disabilities	Explores the diagnostic criteria, characteristics, and classification of mathematical learning disabilities.	18	29.0%
Neurobiological Basis of Mathematical Learning Disabilities	Addresses the neurobiological basis of mathematical learning disabilities, including abnormalities in brain structure and function.	26	41.9%
Intervention and Educational Strategies for Mathematical Learning Disabilities	Explores how different educational and psychological intervention strategies can assist individuals with mathematical learning disabilities.	39	62.9%
Relationship Between Mathematical Anxiety and Cognitive Function	Focuses on how mathematical anxiety impacts individual cognitive functions such as working memory, attention, and executive functions.	13	21.0%

Through the comprehensive analysis and summary of these literatures, we can gain a deeper understanding of the neuroscience basis of mathematical anxiety and cognitive impairment and how to apply this knowledge in educational practices. This not only helps us understand the nature of mathematical anxiety and cognitive impairment more comprehensively but also assists us in finding more effective assessment and intervention methods.

## 3 Results

This review aims to explore the neural foundations of mathematical cognition, mathematical cognitive impairment, and math anxiety, as well as the implications of neuroscience research findings for math education.

### 3.1 Neural foundations of mathematical cognition

Mathematical cognition, as a complex psychological process, involves activities in multiple brain regions. While studies have indicated associations between different components of mathematical cognition and specific brain regions, it’s crucial to note that these relationships are not always straightforward or one-to-one. For instance, while number representation and processing, arithmetic rules and calculations, and problem-solving have been linked to distinct cognitive mechanisms and brain activities (Newman et al., 2011; Bulthé et al., 2014; Park et al., 2014), Cappelletti et al. (2012) found that most patients with focal brain lesions exhibited calculation deficits but did not necessarily show deficits in nonsymbolic quantity recognition. This was observed both in patients with parietal lesions and those with lesions in other brain areas, suggesting a more intricate relationship between brain regions and numerical cognition than previously assumed.

### 3.2 Mathematical cognitive impairment

Mathematical cognitive impairment is a complex phenomenon, covering two main areas: developmental calculation disorders and acquired calculation disorders. Developmental calculation disorders typically manifest during the natural developmental process of children, appearing as difficulties in number and calculation abilities without evident neural system damage or specific brain region developmental delays (Lu et al., 2020). These difficulties extend beyond mathematical calculations, including number recognition, comparison, and estimation, possibly due to fundamental neurobiological defects such as working memory, attention, and executive function deficits (Li et al., 2013; Bulthé et al., 2019; Klados et al., 2019). Acquired calculation disorders, on the other hand, result from external factors like brain injuries or diseases, leading to the loss or decline of previously acquired mathematical abilities (Siemann and Petermann, 2018), typically emerging in adulthood and potentially affecting individuals’ daily lives and career development.

### 3.3 Math anxiety

Math anxiety refers to the tension and unease experienced by individuals when facing mathematical tasks. This anxiety can negatively impact the completion of mathematical tasks, such as reducing task accuracy and efficiency (Foley et al., 2017). Math anxiety may be related to individuals’ self-evaluation, math self-efficacy, and past math experiences. Research has found that math anxiety is associated with activities in specific brain regions, such as the amygdala (Atabek et al., 2022). Individuals with high math anxiety show excessive activity in the amygdala region of the brain when facing mathematical tasks, indicating that math anxiety is not only a psychological phenomenon but also related to brain physiological activities. Math anxiety affects mathematical cognition, including number representation, calculation, and problem-solving (Suárez-Pellicioni et al., 2013; Peña and Suárez-Pellicioni, 2014; Klados et al., 2015). For instance, math anxiety might interfere with individuals’ working memory, making them more likely to feel confused and lose focus when solving mathematical problems. Additionally, math anxiety may also affect individuals’ math learning and long-term math achievements (Peña et al., 2019).

### 3.4 Interaction between math anxiety and cognitive impairment

There is a close interaction between math anxiety and cognitive impairment. Math anxiety might exacerbate the manifestations of cognitive impairment and vice versa (Devine et al., 2018). For example, math anxiety might affect individuals’ attention allocation and information processing strategies, thereby affecting their mathematical cognition performance (Pizzie and Kraemer, 2017). At the same time, individuals with cognitive impairments might develop higher math anxiety due to their mathematical ability deficiencies (Young et al., 2012).

### 3.5 Implications of neuroscience research findings for math education

The findings from neuroscience provide new perspectives and theoretical support for math education. For instance, educators can design teaching strategies more aligned with students’ cognitive development based on the neural foundations of mathematical cognition (Tschentscher and Hauk, 2014). Moreover, for mathematical cognitive impairment and math anxiety, educators can formulate more scientific and effective teaching and intervention strategies based on neuroscience research findings (Faramarzi and Sadri, 2014).

In conclusion, math anxiety and cognitive impairment play significant roles in mathematical cognition. Understanding their neural foundations and mechanisms of mutual influence is crucial for optimizing math education strategies and improving teaching outcomes. In the subsequent discussion section, we will explore issues related to mathematical cognitive impairment and math anxiety more deeply, aiming to provide more comprehensive and profound theoretical support for the improvement and development of math education.

## 4 Discussion

### 4.1 Neural mechanisms of math anxiety

Math anxiety, as a significant research subject in psychology and education fields, has its neurobiological foundations becoming a current research hotspot. Through neuroimaging technologies such as functional magnetic resonance imaging (fMRI), researchers aim to unveil the neural mechanisms and network connections behind math anxiety (Newman et al., 2011). Firstly, from an emotional processing perspective, studies have found that individuals with math anxiety, when facing mathematical tasks, show more active neural activities in brain regions related to emotional processing, such as the amygdala (Atabek et al., 2022). The amygdala, as a core area for emotional responses and processing, indicates that math anxiety is closely related to the brain’s emotional regulation mechanisms (Atabek et al., 2022). This connection might be a crucial neural foundation for the emergence and maintenance of math anxiety. Secondly, math anxiety is also related to the brain’s cognitive control areas. Research indicates that in individuals with math anxiety, brain regions related to executive functions and attention regulation, such as the prefrontal and parietal lobes, might display activity patterns different from those of normal individuals when processing mathematical tasks (Chen et al., 2006). These differences might lead to issues in cognitive resource allocation and utilization in individuals with math anxiety, thereby affecting task completion efficiency and accuracy. Additionally, math anxiety might also be related to the brain’s default mode network (DMN). Some studies have found that the DMN’s activity might be affected in individuals with math anxiety during mathematical tasks (Pletzer et al., 2015). The DMN is usually active when individuals are at rest and decreases in activity during specific cognitive tasks (Gotlieb et al., 2016). Math anxiety might affect this network’s normal functions, thereby affecting individuals’ cognitive performance. In conclusion, the neural mechanisms of math anxiety might involve multiple brain regions and networks, including those related to emotional processing, cognitive control, and the default mode network (Young et al., 2012; Pletzer et al., 2015; Meijer et al., 2022). This multi-region, multi-network involvement suggests that math anxiety is a complex psychological phenomenon, influenced by various neural factors. A deeper understanding of the neural mechanisms of math anxiety can not only help us comprehend this phenomenon more comprehensively and accurately but also provide more scientific and effective theoretical guidance for clinical interventions and educational practices. For instance, targeted regulation and training of related brain regions and networks might help alleviate the degree of math anxiety, improving individuals’ math learning and performance (Liu et al., 2019). This will contribute to the advancement of math education, enhancing the quality and effectiveness of education.

### 4.2 Relationship between mathematical anxiety and components of mathematical cognition

Math anxiety is a specialized, negative emotional reaction directed towards math learning and evaluation. This emotional reaction is not merely limited to superficial emotional experiences; more profoundly, it is intricately linked to the cognitive components of math—such as number representation, calculation, and problem-solving (Suárez-Pellicioni et al., 2013; Peña and Suárez-Pellicioni, 2014; Klados et al., 2015). Through in-depth analysis and exploration, we can more comprehensively understand how math anxiety permeates and influences an individual’s mathematical cognitive processes.

Firstly, we explore the relationship between math anxiety and number representation. Number representation is foundational to mathematical cognition, involving the cognitive representation of numbers and quantities (Bulthé et al., 2014). Individuals with high math anxiety may encounter difficulties in number representation (Piazza and Eger, 2016). For instance, when engaging in tasks such as number magnitude comparison, number ordering, and number line positioning, they may not perform as well as those with low math anxiety. This could be because the excessive attention and worry triggered by math anxiety interfere with the normal processing and encoding of numerical information, affecting the process of number representation (Kanayet et al., 2018).

Secondly, we examine how math anxiety impacts calculation abilities. Calculation abilities are a core component of mathematical cognition, encompassing basic arithmetic calculations and complex mathematical operations (Yi-Rong et al., 2011; Van Der Ven et al., 2016). Research has found that individuals with high math anxiety may make more errors and take longer reaction times when performing arithmetic calculations (Kanayet et al., 2018). This might be due to math anxiety consuming substantial cognitive resources, such as attention and working memory, preventing individuals from fully concentrating when processing calculation tasks, thereby affecting the accuracy and efficiency of calculations.

Lastly, we investigate the relationship between math anxiety and problem-solving abilities. Problem-solving is an advanced process in mathematical cognition, involving metacognitive skills such as strategy selection, planning, and self-monitoring. Individuals with high math anxiety, when facing mathematical problems, may feel more anxious and uneasy, which could affect their strategy selection and problem-solving processes. For example, they might exhibit more hesitation and uncertainty during problem-solving, and the strategies they choose may not be as effective and reasonable (Peters et al., 2016).

By deeply exploring the relationship between math anxiety and components of mathematical cognition, we can see how math anxiety influences mathematical cognition through various pathways and mechanisms. Math anxiety might indirectly affect the execution of mathematical tasks by occupying limited cognitive resources and disrupting the normal functioning of attention and working memory (Evans et al., 2016). Simultaneously, math anxiety might also directly interfere with the problem-solving process by affecting individuals’ metacognitive skills, such as self-monitoring and strategy selection (Peters et al., 2016).

In conclusion, there exists a complex and multi-layered interaction and influence between math anxiety and the components of mathematical cognition. To more effectively understand and alleviate the negative impacts of math anxiety, future research could further delve into the specific mechanisms and pathways of these interactions, providing more scientific and targeted guidance and recommendations for educational practice.

### 4.3 Manifestations and characteristics of cognitive impairments

In discussing cognitive impairments in mathematical cognition, literature primarily focuses on Developmental Calculation Disorder (DCD) and Acquired Calculation Disorder (ACD) (Kucian et al., 2013; Van Beek et al., 2015). Both types of impairments are mainly characterized by deficiencies in numerical and calculation abilities, but their manifestations and etiologies differ.

#### 4.3.1 Developmental calculation disorder (DCD)

DCD, as a specific learning impairment, predominantly manifests during the mathematical learning process in children. This impairment is usually persistent, affecting individuals’ performance in various areas such as numerical processing, basic arithmetic skills, and mathematical problem-solving (Kaufmann et al., 2011; Kucian et al., 2011; Kucian and Von Aster, 2014). Notably, studies like Demeyere et al. (2010) and Demeyere et al. (2012) have highlighted dissociations between components like subitizing and counting in patients with dyscalculia. Furthermore, the work of Brian Butterworth has extensively delved into the intricacies of mathematical cognition and its disorders. While DCD is often associated with unusual patterns of brain functioning, particularly involving the parietal lobes, it is rarely the result of brain damage and is unlikely to have an exact parallel with acquired dyscalculia (ACD). There is still controversy about the exact nature and causes of DCD (Murphy et al., 2007; Kaufmann et al., 2013; Träff et al., 2017). A detailed exploration and analysis of DCD manifestations, based on literature, will be conducted (see Table 3) (Van Harskamp and Cipolotti, 2001; Kaufmann et al., 2011; Kucian et al., 2011; Faramarzi and Sadri, 2014; Kucian and Von Aster, 2014).

**Table 3:** Manifestations of developmental dyscalculia.

Domain	Subdomain	Difficulties experienced by children with DCD
Numerical Processing	Number Recognition	More time needed for number recognition, errors may occur during the process
	Numerical Magnitude Representation	Difficulties in accurately comparing and estimating numbers, challenges in number sorting and categorization
Basic Arithmetic Skills	Arithmetic Rules and Strategies	Deficiencies in mastering and applying basic arithmetic rules and strategies
	Calculation Efficiency	Reliance on finger and physical counting, lack of effective calculation strategies and techniques
Problem-Solving Ability	Problem-Solving Strategies	Lack of effective and flexible problem-solving strategies
	Efficiency and Accuracy in Problem-Solving	Lower efficiency, higher error rates, confusion, and frustration when facing complex and novel problems

Through an in-depth analysis of DCD children in terms of numerical processing, basic arithmetic skills, and mathematical problem-solving abilities, we can more comprehensively and accurately understand the mathematical learning characteristics and difficulties of this group. This is crucial for educators and mental health professionals to provide more precise and personalized support during diagnosis and intervention. Moreover, it helps further explore and understand the causes and mechanisms of mathematical learning impairments, providing a richer and more profound theoretical foundation for future research and practice.

#### 4.3.2 Acquired calculation disorder (ACD)

ACD, as a unique form of mathematical cognitive impairment, is typically due to brain injury or neurological disorders (Siemann and Petermann, 2018). Unlike DCD, individuals with ACD might have successfully acquired certain mathematical skills earlier, but due to subsequent factors such as brain injury or disease, these skills may be lost or deteriorated (Miundy et al., 2019). The main manifestations and characteristics of ACD, such as loss of mathematical skills, calculation difficulties, and mathematical thinking impairments, will be deeply explored.

Loss of Mathematical Skills:Individuals with ACD may lose some basic mathematical skills that they had previously mastered, including basic arithmetic abilities, understanding, and application of mathematical concepts (González et al., 2019). They might find simple arithmetic operations challenging or feel confused when understanding and applying basic mathematical concepts and formulas. This loss may affect their mathematical application abilities in daily life and learning, such as calculating shopping expenses and measuring object lengths and areas (Benavides-Varela et al., 2017).

Calculation Difficulties:Due to brain injuries, individuals with ACD may face significant challenges in mathematical calculations (Cohen et al., 2018). This includes not only complex mathematical calculations, such as solving algebraic and geometric problems, but also simpler tasks like basic arithmetic operations (Kaufmann, 2008; Kunwar, 2021). They might make mistakes easily during calculations or take longer to complete tasks that should be simple and quick.

Mathematical Thinking Impairments:Individuals with ACD may also have certain limitations in mathematical thinking and reasoning (Siemann and Petermann, 2018). They might display rigidity, lack of creativity, and flexibility when facing mathematical problems (Claros-Salinas et al., 2014; Hobri et al., 2021). They might find it difficult to understand and master new mathematical concepts and methods or lack effective problem-solving strategies and methods. Additionally, they might struggle with abstract thinking and logical reasoning, such as understanding and applying abstract mathematical concepts and theorems.

In conclusion, ACD, as a special form of mathematical cognitive impairment, is mainly characterized by the loss of mathematical skills, calculation difficulties, and mathematical thinking impairments. These impairments may severely affect the mathematical application abilities and performance of individuals with ACD in learning, work, and daily life (Kaufmann et al., 2013). Therefore, more attention and support are needed for individuals with ACD, helping them overcome obstacles and improve mathematical cognition and application abilities through effective educational and rehabilitative interventions.

By analyzing developmental and acquired calculation disorders, it is evident that although both are related to deficiencies in mathematical cognitive abilities, their causes, manifestations, and impacts are distinct. Understanding and distinguishing the characteristics of these impairments are essential for better understanding the potential issues in mathematical cognitive processes and providing more precise and effective assistance to individuals facing difficulties.

### 4.4 Neural basis of cognitive impairments

In the profound exploration of cognitive impairments, scholars have utilized sophisticated neuroimaging technologies such as Diffusion Tensor Imaging (DTI), conducting a series of exhaustive investigations into the neural foundations of cognitive impairments (Kucian et al., 2013). These explorations aim to unveil the neural structural and functional abnormalities underlying the difficulties encountered by individuals with cognitive impairments during mathematical tasks (Murphy et al., 2007).

Initially, research has revealed that individuals with cognitive impairments may exhibit structural and functional anomalies in critical brain regions such as the parietal and frontal lobes (Kaufmann et al., 2011; Bulthé et al., 2019). These lobes, essential components of the brain, play central roles in cognitive processes such as spatial representation, attention, memory, and executive functions (Kaufmann et al., 2011). In the parietal lobe, abnormalities may be related to difficulties in spatial representation and visual–spatial processing, crucial for understanding and solving geometric and spatially related mathematical problems (Bulthé et al., 2019). In the frontal lobe, anomalies might predominantly affect executive functions, including planning, organization, and self-monitoring, essential elements in mathematical problem-solving (Kaufmann et al., 2011).

Furthermore, utilizing neuroimaging technologies like DTI, researchers have discovered potential abnormalities in the white matter pathways of individuals with cognitive impairments (Jolles et al., 2015). White matter pathways, the “highways” for neural information transmission between various brain regions, are vital for ensuring coordination and integration among different functional networks of the brain (Kucian et al., 2013). In individuals with cognitive impairments, abnormalities in these pathways might lead to reduced efficiency in information transmission, thereby affecting the processing and integration of mathematical information. Additionally, these neural foundation abnormalities might be directly linked to the specific manifestations of individuals with cognitive impairments in mathematical tasks (Davidse et al., 2014). For instance, anomalies in the parietal and frontal lobes might make it challenging for individuals to effectively organize and utilize relevant strategies and knowledge during mathematical calculations and problem-solving. Abnormalities in the white matter pathways might affect the mobilization and utilization of various cognitive resources when handling complex mathematical tasks (Grant et al., 2020).

These research findings offer invaluable perspectives, aiding in a more comprehensive and profound understanding of the neural mechanisms of cognitive impairments in mathematical learning. This not only facilitates the enhancement of precision in the diagnosis and assessment of cognitive impairments but also provides robust theoretical support for devising effective educational intervention measures and strategies. A deeper understanding of the neural basis of cognitive impairments allows for the development of more targeted teaching methods and strategies that align with the characteristics of individuals with cognitive impairments, aiming to better support their development and progress in mathematical learning.

### 4.5 Research on intervention strategies

In exploring intervention strategies for mathematical anxiety and cognitive impairments, researchers have employed various methods, aiming to find effective ways to alleviate individuals’ mathematical anxiety and improve their mathematical cognitive abilities.

#### 4.5.1 Cognitive training interventions

Recently, cognitive training has emerged as a crucial intervention strategy, extensively researched and applied to alleviate mathematical anxiety and improve cognitive impairments. To enhance individuals’ cognitive abilities and information processing efficiency in mathematical learning, researchers have meticulously designed a series of systematic cognitive tasks. Among them, working memory training is a core component of cognitive training, involving the cognitive system where people temporarily store and manipulate information, directly affecting mathematical learning outcomes (De Vreeze-Westgeest and Vogelaar, 2022). Researchers, through designing tasks of various forms and difficulties such as n-back tasks and complex span tasks, intentionally enhance individuals’ working memory capabilities (Schmidt et al., 2009; Lucidi et al., 2014). With continuous and regular training, individuals can process and manipulate information more effectively in mathematical tasks, reducing the cognitive load caused by mathematical anxiety.

Cognitive training also includes attention control training, helping individuals selectively focus on and process task-related information while ignoring irrelevant distractions (Bishara and Kaplan, 2021). Through specialized training such as the Flanker task and Stroop task, individuals learn to concentrate better, reducing attention dispersion and cognitive resource consumption caused by anxiety (Van Der Ven et al., 2011; Van Nes, 2011; Peralbo et al., 2020). Additionally, cognitive training emphasizes enhancing other cognitive functions such as executive functions and spatial abilities (Wu et al., 2019; Chatzivasileiou and Drigas, 2022). These trainings assist individuals in more flexibly and accurately applying various strategies and methods during the mathematical learning process, improving problem-solving abilities. During the implementation of cognitive training, researchers particularly emphasize the individualization and adaptability of training, adjusting the difficulty and content of training tasks timely based on each individual’s baseline abilities and progress, ensuring the effectiveness and efficiency of the training (Chipman, 2010).

In conclusion, cognitive training, as an intervention strategy based on cognitive psychology principles, shows immense potential in alleviating mathematical anxiety and improving cognitive impairments by specifically training cognitive functions such as working memory and attention control. Looking forward, research can further explore the optimal implementation methods and effectiveness evaluation approaches of cognitive training, providing more scientific and effective guidance and strategies for cognitive training-based interventions.

#### 4.5.2 Application of psychological interventions

The significance of psychological interventions in alleviating mathematical anxiety has been widely affirmed by extensive research. As an effective strategy to mitigate mathematical anxiety, psychological interventions not only assist individuals in altering their thought processes and emotional responses, thereby reducing the levels of mathematical anxiety, but also enhance individuals’ confidence and efficiency in mathematical learning. Cognitive-behavioral therapy, psychoeducation, and relaxation training are currently the three mainstream psychological intervention methods (Henschel and Roick, 2020; Moustafa et al., 2021; Ng et al., 2022).

Research indicates that cognitive-behavioral therapy (CBT) is a promising strategy. This approach focuses on helping individuals identify and challenge their negative and irrational thinking patterns, encouraging students to assess mathematical tasks and challenges more objectively, thus alleviating anxiety caused by excessive worry and fear (Moustafa et al., 2021). In the long term, this method can enable students to maintain calmness and rationality when facing mathematical challenges, thereby improving mathematical abilities (Ramirez et al., 2018). Psychoeducation is also an essential intervention measure. It aims to enhance students’ understanding of mathematical anxiety, allowing them to better comprehend the causes, characteristics, and impacts of anxiety (Casad et al., 2015). Studies have shown that psychoeducation can help students develop a positive and healthy learning attitude, thus reducing the fear of mathematics (Cheng et al., 2022). Through psychoeducation, students can address mathematical anxiety specifically, establishing a positive emotional connection with mathematics. Relaxation training, on the other hand, starts from a physical perspective, assisting individuals in alleviating the physical and psychological stress generated by mathematics. For instance, techniques such as deep breathing and progressive muscle relaxation have been found to effectively help students maintain psychological balance and reduce tension and anxiety during mathematical learning (Ng et al., 2022).

In conclusion, by integrating the above three strategies, we can assist students in reducing the levels of mathematical anxiety from multiple dimensions. Continuous and systematic psychological interventions are key, enabling individuals to gradually overcome mathematical anxiety and engage in mathematical learning and practice with more confidence and efficiency.

#### 4.5.3 Personalized intervention strategies

In exploring intervention methods for mathematical anxiety and cognitive impairments, personalized intervention strategies have emerged as a focal point receiving significant attention. This strategy emphasizes developing and implementing intervention plans based on each learner’s unique characteristics and needs, aiming to provide more precise and targeted assistance.

Firstly, this strategy is based on a clear premise: each learner exhibits variations in cognitive abilities and mathematical anxiety (Li et al., 2021). Research suggests that to effectively address specific problems encountered by learners in mathematical learning, intervention plans must consider the type and degree of learners’ cognitive impairments and the manifestation and severity of mathematical anxiety (Luneta and Sunzuma, 2022). Therefore, intervention plans should be meticulously tailored to learners’ specific situations. Secondly, personalized intervention strategies are not merely preliminary planning but represent a dynamic, continuously adjusting process. Based on learners’ feedback and progress during the intervention, strategies and methods will be timely adjusted to ensure they consistently meet learners’ actual needs (Johnson et al., 2020). The flexibility of this strategy not only ensures that interventions always align with learners’ needs but also enhances learners’ participation and acceptance. Additionally, intervening solely from mathematical anxiety and cognitive perspectives is insufficient. Personalized intervention strategies emphasize a comprehensive focus on learners, providing help not only from a cognitive perspective but also considering learners’ psychological, social, and emotional factors, offering holistic support (Reyes, 2019; Shafiq et al., 2021). For example, enhancing learners’ self-efficacy and learning motivation, optimizing the learning environment, and strengthening social support are all considerations within this strategy.

In general, personalized intervention strategies emphasize respect for individual differences and support for holistic development, offering effective assistance to learners encountering anxiety and cognitive impairments in mathematical learning. Through the implementation of this strategy, we hope to assist learners in achieving greater progress and development in mathematical learning.

#### 4.5.4 Integrated intervention methods

Integrated intervention methods have increasingly attracted attention as an innovative strategy in the research field of mathematical anxiety and cognitive impairments. Research indicates that this method, by combining cognitive training with psychological interventions, forms a diversified intervention framework aimed at comprehensively improving individuals’ mathematical anxiety and cognitive impairments (Soares et al., 2018). Cognitive training focuses on enhancing the efficiency and accuracy of information processing, including training in working memory, executive functions, and attention control, which helps improve performance in mathematical tasks and reduce the cognitive load brought about by mathematical anxiety (Schmidt et al., 2009; Wu et al., 2019; Chatzivasileiou and Drigas, 2022). Psychological interventions focus on emotional management and regulation, helping individuals identify, understand, and regulate negative emotions and thoughts related to mathematical anxiety through methods such as cognitive-behavioral therapy and relaxation training (Henschel and Roick, 2020; Moustafa et al., 2021; Ng et al., 2022). The advantage of integrated intervention methods lies in their comprehensiveness, enabling interventions from both cognitive and emotional dimensions. This strategy helps address the issues of mathematical anxiety and cognitive impairments more effectively. Through integrated interventions, individuals can not only enhance their cognitive abilities but also achieve better management and regulation at the emotional level. Moreover, this method emphasizes individualization and flexibility, allowing for the adjustment and combination of different intervention strategies based on individuals’ specific needs, aiming to achieve optimal intervention outcomes. In conclusion, integrated intervention methods provide a new, diversified strategy and approach for the intervention of mathematical anxiety and cognitive impairments. Future research is expected to further explore and optimize this intervention method, hoping to achieve better intervention outcomes in practical applications.

#### 4.5.5 Evaluation of intervention effects

In psychological and educational intervention research, there are various essential methods to evaluate intervention effects. Research indicates that a direct comparison of performance before and after intervention is one of the fundamental methods to evaluate intervention effects, helping to measure changes in relevant indicators such as the level of mathematical anxiety and cognitive abilities (Casad et al., 2015). Meanwhile, comparison with a control group is considered a more precise evaluation method. Through this comparison, the effect of the intervention can be judged more accurately, eliminating other possible interfering factors. Additionally, the importance of long-term tracking should not be overlooked (Bishara and Kaplan, 2021). Long-term tracking can help researchers explore the sustainability and stability of intervention effects, providing a more accurate evaluation of the long-term effects of intervention strategies (Ng et al., 2022). Research should also consider the multidimensionality of evaluation. Multidimensional evaluations, including assessments of individuals’ self-efficacy, motivation, and emotional regulation abilities, can provide a more comprehensive and profound understanding of the effects of intervention strategies (Chipman, 2010). By integrating various evaluation methods, a more comprehensive and accurate conclusion can be drawn. In summary, evaluating intervention effects is a complex and multi-level process. Through meticulous and comprehensive evaluation, we can better understand and validate the effectiveness of intervention strategies, providing robust support and valuable references for future research and practice.

In conclusion, intervention strategies for mathematical anxiety and cognitive impairments are diversified and comprehensive. Through continuous research and exploration, we can continually refine and optimize intervention methods, providing more effective support for alleviating mathematical anxiety and improving cognitive impairments.

### 4.6 Comparison of research methods and results

In the in-depth exploration of the interactive relationship between mathematical anxiety and cognitive impairments, we must confront a reality: significant disparities exist in the research methods and results across various studies. These differences offer us a valuable opportunity to understand this complex phenomenon from multiple angles and dimensions. Below is a detailed comparison and analysis of the research methods and results from various studies.

#### 4.6.1 Diversity of research focus

In exploring the intertwined relationship between mathematical anxiety and cognitive impairments, research exhibits a rich diversity of focuses. Firstly, some studies predominantly concentrate on the neural mechanisms of mathematical anxiety and cognitive impairments, often employing advanced neuroimaging techniques such as functional magnetic resonance imaging (fMRI) and diffusion tensor imaging (DTI) (Newman et al., 2011; Jolles et al., 2015). These studies reveal how mathematical anxiety affects individuals’ cognitive processing at the neural level, providing neurobiological evidence for a deeper understanding of the nature of mathematical anxiety and its impact on individuals’ mathematical learning abilities. For instance, studies have found that individuals with mathematical anxiety exhibit increased activity in brain regions associated with emotional regulation and decreased activity in regions related to mathematical information processing when handling mathematical tasks (Young et al., 2012). Exploring these neural mechanisms contributes to a deeper understanding of the intrinsic connections between mathematical anxiety and cognitive impairments. On the other hand, some studies focus more on the design and evaluation of intervention strategies, seeking and validating methods to alleviate mathematical anxiety and improve cognitive impairments (Schmidt et al., 2009; Wu et al., 2019; Li et al., 2021; Chatzivasileiou and Drigas, 2022; Ng et al., 2022). For example, by implementing cognitive-behavioral therapy or psychoeducational interventions, these studies aim to reduce students’ levels of mathematical anxiety and help improve their mathematical learning outcomes (Bishara and Kaplan, 2021; Moustafa et al., 2021). Research focused on interventions holds practical value and guidance, offering us methods to practically address and improve mathematical anxiety and cognitive impairments.

In consideration of the above, the diversity of research focuses enriches our understanding of the relationship between mathematical anxiety and cognitive impairments. It provides approaches and methods to address and study this issue from different angles and dimensions, contributing to a more comprehensive and profound exploration of the complex relationship between mathematical anxiety and cognitive impairments.

#### 4.6.2 Variability in sample selection

In discussing the relationship between mathematical anxiety and cognitive impairments, the strategies and methods of sample selection hold decisive importance. Through a comprehensive analysis of multiple studies, we find significant variability in sample selection, directly affecting the depth, breadth, and representativeness of the research conclusions.

Firstly, some studies show that researchers have a clear focus on sample selection, specifically choosing participants from certain age groups or backgrounds, such as elementary and high school students, or university students. This targeted selection provides researchers with a more focused perspective, enabling them to delve deeply into the manifestations of mathematical anxiety in specific groups (Zhou et al., 2007; Prado et al., 2014; Jolles et al., 2015). For example, some studies have found that middle school students have higher levels of mathematical anxiety compared to university students, possibly related to their academic pressures and external expectations (Klados et al., 2015). This research method helps accurately reveal the characteristics of mathematical anxiety at different educational stages. However, other studies have adopted a broader sample selection, encompassing various ages, genders, cultures, and educational backgrounds, thus making the research results more universal and representative (Yi-Rong et al., 2011; Jolles et al., 2015). Such a method can help us understand the manifestations of mathematical anxiety in various populations more comprehensively, thereby revealing its general connections with cognitive impairments. More importantly, this broad sample selection provides an opportunity to explore the potential influences of background factors such as culture and education on mathematical anxiety, offering robust guidance for the formulation of effective intervention measures in the future.

In conclusion, the variability in sample selection not only affects the focus and depth of the research but also determines the application and interpretation of the research results. Therefore, future research should pay more attention to sample selection strategies to reveal the relationship between mathematical anxiety and cognitive impairments more precisely, providing more practical references for educational and psychological interventions.

#### 4.6.3 Diversity of data analysis methods

In the relevant research exploring the interactive relationship between mathematical anxiety and cognitive impairments, the diversity of data analysis methods has emerged as a significant characteristic. Many scholars have adopted quantitative analysis methods, utilizing rigorous statistical tests such as t-tests, analysis of variance, and regression analysis to validate research hypotheses, and quantitatively assess the relationship between mathematical anxiety and cognitive impairments based on large sample sizes (Rosenberg-Lee et al., 2011; Soltész et al., 2011; Klados et al., 2017; Choi-Koh and Ryoo, 2019). These studies present results in the form of data and charts, providing intuitive evidence for unveiling the objective relationship between mathematical anxiety and cognitive impairments. Simultaneously, some studies have chosen qualitative analysis methods, collecting data through open-ended questionnaires, in-depth interviews, and focus group discussions, to explore and describe individuals’ intrinsic experiences and feelings when facing mathematical learning more profoundly (Young et al., 2012; Batashvili et al., 2019; Peña et al., 2019). This approach focuses on revealing students’ genuine reactions and feelings in mathematical learning, providing rich background information for understanding how mathematical anxiety leads to cognitive impairments. Some studies have adopted mixed methods, integrating both quantitative and qualitative analyses, aiming to reveal the interactive relationship between mathematical anxiety and cognitive impairments more comprehensively (Rosenberg-Lee et al., 2011; Young et al., 2012; Peña et al., 2019). This strategy helps obtain a rich diversity of data, allowing for a more comprehensive and profound understanding and interpretation of how mathematical anxiety affects individuals’ cognitive processes.

Considering the diversity of these research methods reflects that scholars are exploring the issue of mathematical anxiety and cognitive impairments from various angles and dimensions. This methodological diversity enriches the research content and helps us understand the complex relationship between mathematical anxiety and cognitive impairments more comprehensively and profoundly, allowing for a better grasp of its essence and patterns.

#### 4.6.4 Interpretation and understanding of research results

Through a comprehensive analysis of numerous literature, we find significant disparities in the interpretation and understanding of research on mathematical anxiety and cognitive impairments, mainly reflected in the emphasis on individual differences and the exploration of general rules and mechanisms.

Some studies indicate that individuals, when facing mathematical tasks, exhibit varying degrees of mathematical anxiety and cognitive impairments due to differences in personal experiences and cognitive processing methods (Bulthé et al., 2019). These studies emphasize proposing targeted intervention measures based on individuals’ specific situations. For example, providing psychological counseling for individuals who have mathematical anxiety due to past failures, and offering learning guidance and strategy training for those lacking effective learning strategies. On the other hand, some studies focus more on exploring the general rules and mechanisms of mathematical anxiety and cognitive impairments, trying to find universal solutions to guide teaching and intervention (Li et al., 2013; Foley et al., 2017; Klados et al., 2019). For instance, enhancing students’ self-efficacy and autonomous learning abilities is considered an effective way to alleviate mathematical anxiety and improve cognitive impairments.

To achieve more comprehensive and effective teaching and intervention, future research should find a balance between individual differences and general rules. We should consider each student’s uniqueness, providing personalized support that meets their needs, while also exploring the common rules of mathematical anxiety and cognitive impairments, forming systematic and scientific teaching and intervention strategies. This integrated approach helps students overcome mathematical anxiety and cognitive impairments more effectively, thereby improving the effectiveness and quality of mathematical learning.

In conclusion, the differences in research methods and results across various literature provide us with a valuable perspective to understand the interactive relationship between mathematical anxiety and cognitive impairments from multiple angles and dimensions. This diversity and complexity require us to consider various factors comprehensively when analyzing and comparing, allowing for a more comprehensive and profound understanding of the current research status and development trends in this field.

### 4.7 Limitations

In the current academic environment, significant progress has been made in the research on mathematical cognitive impairments and math anxiety. However, as with many academic studies, research in this field also has its inherent limitations. Below is a detailed discussion of these limitations:

#### 4.7.1 Issue of sample size

For studies in the neuroscience field exploring the relationship between math anxiety and cognitive processes, the size and representativeness of the sample constitute significant limitations.

Firstly, a smaller sample might lead to Type II errors, where an actual effect is not detected due to insufficient sample size. Such limitation could cause researchers to erroneously conclude that there is no association between math anxiety and a particular cognitive process when, in fact, such an association might exist. Additionally, another notable limitation of a small sample is its impact on the replicability of research results. Specific samples might make the results too dependent on that sample, questioning the applicability in a broader population. Secondly, the representativeness of the research sample is another critical limitation. If the research sample lacks diversity, such as being limited to specific age groups, genders, or cultural backgrounds, it might make the research results less universally applicable. For instance, studies involving only university students might not be generalizable to other age groups, constituting its limitation. Practical research conditions and constraints, such as funding, time, and technology, often further exacerbate these limitations. High costs and technical difficulties might limit the number and diversity of recruitable samples, especially in studies involving high-cost technologies such as functional magnetic resonance imaging (fMRI) and functional near-infrared spectroscopy (fNIRS) (Artemenko et al., 2019; Shi et al., 2023).

In conclusion, despite the unique value of neuroscience research, the limitations in sample size and representativeness need full recognition and attention. Researchers should consider these limitations when assessing the reliability and universality of their research results and seek methods to address or mitigate these issues where possible.

#### 4.7.2 Diversity of research methods

In the research on math anxiety and cognitive impairments, a variety of research methods have been employed, including functional magnetic resonance imaging (fMRI), event-related potentials (ERP), behavioral assessment tools, and functional near-infrared spectroscopy (fNIRS). Each of these methods has its unique advantages and limitations, collectively constituting a rich research toolbox, allowing for an in-depth exploration of such complex phenomena from multiple angles.

Firstly, fMRI, with its high spatial resolution, can capture real-time brain activity during mathematical tasks, but its main limitation lies in the inability to directly measure neuronal activity, and it is relatively costly (Holloway et al., 2013). ERP, with its high temporal resolution, can measure the brain’s immediate response to specific stimuli or events, but its spatial resolution is lower, and the source of signals might be challenging to determine (Klados et al., 2015). Behavioral assessment tools are relatively simple, low-cost, and suitable for large-sample research but might not fully reveal individuals’ intrinsic cognitive and emotional processes.

However, the diversity of these methods also brings challenges to research. Different research methods might lead to different interpretations and conclusions regarding the same phenomenon. For example, an fMRI-based study might reveal that a particular brain area is more active in individuals with math anxiety (Newman et al., 2011), while an ERP-based study might find that this activity is related to a specific cognitive process (Peña and Suárez-Pellicioni, 2014). Moreover, since each method has its inherent limitations, such as dependency on technology and equipment, influence of experimental conditions and analysis methods, and interference of cultural, educational backgrounds, and individual differences, researchers need to apply multiple methods comprehensively to understand math anxiety and cognitive impairments more accurately and thoroughly. Through cross-validation and integrated analysis, more in-depth and comprehensive research results are expected to be obtained.

#### 4.7.3 Limitations in research focus

In the field of research on mathematical cognitive impairments and math anxiety, the current focus predominantly lies on fundamental mathematical skills, such as basic computational abilities and numerical representation (Rosenberg-Lee et al., 2011; Prado et al., 2014; Jolles et al., 2015). The limitation of this focus manifests as a relative neglect of more advanced and complex mathematical cognitive processes, such as abstract thinking, problem-solving, and logical reasoning. This not only may constrain a more comprehensive and profound understanding of math anxiety and mathematical cognitive impairments but also may adversely impact the design and implementation of educational practices and intervention strategies.

Firstly, while fundamental mathematical skills are the basis of mathematical learning, focusing solely on them might overlook the diversity and complexity of mathematical cognition. For instance, abilities like abstract thinking and problem-solving are essential for understanding and mastering mathematical concepts, conducting mathematical reasoning, and solving complex problems. Secondly, the current research focus might also limit a more comprehensive exploration of math anxiety and mathematical cognitive impairments, as these issues are related not only to basic skills but also to more advanced mathematical cognitive processes.

Moreover, an excessive focus on fundamental skills might lead to a monotonous and one-sided orientation in educational and intervention strategies, affecting their comprehensive effectiveness. For more effective promotion of mathematical learning and alleviation of math anxiety, research and practice need to consider the comprehensiveness and diversity of mathematical cognition.

In conclusion, research should pay balanced attention to both fundamental skills and advanced mathematical cognitive processes, to promote comprehensive theoretical and practical development in the field of mathematical cognitive impairments and math anxiety. Future research needs to explore more deeply various aspects of mathematical cognition, including complex and advanced cognitive processes beyond basic skills. This way, we can understand math anxiety and mathematical cognitive impairments more comprehensively and provide richer and more comprehensive guidance for educational practice and intervention strategies.

#### 4.7.4 Issues in interdisciplinary integration

Researching the complex and multifaceted topic of mathematical cognitive impairments and math anxiety involves the intersection of numerous disciplines such as psychology, education, and neuroscience. Although each discipline provides unique and rich perspectives, efficiently integrating these diverse resources has become a core challenge. The primary issue lies in the independent characteristics and focuses of the research methods and theoretical frameworks of each discipline. Psychology mainly studies individuals’ psychological and behavioral responses, neuroscience focuses on exploring biological mechanisms and neural networks, and education emphasizes the effects of teaching methods and educational environments on individuals. This requires researchers to meticulously adjust and integrate the features of each discipline when designing and implementing research. Secondly, successful interdisciplinary research requires researchers to possess high levels of collaboration and communication skills. This not only demands them to understand and master the core knowledge and methods of each discipline but also to establish effective communication and cooperation with peers from different disciplinary backgrounds, undoubtedly setting higher requirements for researchers’ professional competence and interdisciplinary collaboration abilities. Moreover, finding a balance to maintain the uniqueness of each discipline while achieving effective integration is also crucial. Excessive integration might weaken the core advantages of a discipline, while insufficient integration might lead to one-sided or limited research results.

In conclusion, we recognize that interdisciplinary integration in the research of mathematical cognitive impairments and math anxiety is both an essential direction and a challenging task. To obtain more comprehensive and profound research results in this field, we must innovate in theory and methods and strengthen communication and cooperation between disciplines.

#### 4.7.5 Lack of intervention strategies

In the field of research on math anxiety and cognitive impairments, despite the accumulation of rich theoretical knowledge and empirical data, the effective transformation of this knowledge into practical intervention strategies remains a challenge. Currently, although there is a deeper description and explanation of math anxiety and cognitive impairments, practical intervention strategies, whether at the educational, psychological, or neurobiological levels, still appear relatively weak. This poses significant constraints on the application in actual education and psychological health fields.

Firstly, most studies focus on the manifestations and causes of math anxiety and cognitive impairments, with less involvement in specific intervention strategies and methods (Li et al., 2013; Foley et al., 2017; Klados et al., 2019). This necessitates further empirical research to explore and confirm the actual effects of various intervention strategies. Secondly, even if some strategies achieve preliminary effects in the short term, their sustainability and stability remain unknown due to the lack of long-term follow-up evaluations. Future research must pay more attention to evaluating the long-term effects of intervention strategies and exploring how these strategies can be sustainably applied in daily environments. Moreover, given the significant variations in math anxiety and cognitive impairments among individuals, intervention strategies also need to have a certain level of individualization and customization. How to adjust and optimize strategies based on individual differences will be key in future research. Additionally, the implementation of intervention strategies also faces various practical challenges, such as resource allocation, professional training, and integration with current education and mental health services. Solving these challenges requires interdisciplinary efforts and cooperation.

In conclusion, despite a deeper understanding of math anxiety and cognitive impairments, there are still many difficulties to overcome in the research and application of intervention strategies. It is hoped that future research can provide more guiding and practical strategies and methods for this field.

#### 4.7.6 Influence of cultural and social background

When exploring math anxiety and mathematical cognitive impairments, the role of cultural and social backgrounds is indispensable. Both play a key role in determining individuals’ cognitive development and emotional experiences in the mathematical learning process. However, current research mainly focuses on specific cultural and social environments, providing us with limited perspectives (Yi-Rong et al., 2011; Jolles et al., 2015).

Cultural backgrounds profoundly influence the shaping of mathematical cognition, reflected in different cultures’ educational methods, educational emphases, and resource allocations. For instance, some cultures might value basic mathematical skills more, while others might emphasize problem-solving and critical thinking abilities. This implies that the formation of mathematical cognition might vary across different cultures. Social backgrounds also significantly impact math anxiety and mathematical cognitive impairments. They determine societal expectations of mathematical abilities, the social value of mathematical learning, and the general acceptance of math anxiety. For example, some societies might view mathematics as a key competence, while others might not. Furthermore, cultural and social backgrounds also affect how individuals perceive and cope with math anxiety and mathematical cognitive impairments. In some cultures, people might be more willing to openly discuss their difficulties, while in others, they might choose to remain silent about these issues.

In summary, to deeply understand math anxiety and mathematical cognitive impairments, we need to consider a broader cultural and social background. Future research should pay more attention to the diversity of these factors, which can not only provide us with a deeper understanding but also help in formulating more effective and targeted intervention strategies.

In conclusion, although some progress has been made in the research of mathematical cognitive impairments and math anxiety, there are still many unresolved issues and challenges. These limitations remind us to be cautious in interpreting and applying research results and provide direction for future research.

### 4.8 Future research directions

Based on the review and analysis of existing literature, it is evident that math anxiety and cognitive impairments are two important and interconnected research focuses in the field of mathematical cognition. Future research can further expand and deepen in the following directions:

#### 4.8.1 In-depth study of math anxiety

(1) Exploration of neural mechanisms

To deeply understand the neural mechanisms behind math anxiety, future research should focus on revealing how neurotransmitter changes and synaptic plasticity influence the onset and maintenance of math anxiety. Current research has somewhat demonstrated the association between math anxiety and brain activity patterns through functional brain imaging technologies, but a comprehensive understanding of the neurobiological foundations is still pending. Neurotransmitters such as serotonin, dopamine, and cortisol may play crucial roles in math anxiety. Therefore, there should be a profound exploration of how these neurotransmitters influence the levels of math anxiety and whether they can serve as potential targets for alleviating math anxiety. Additionally, research should assess whether there are abnormalities in the synaptic plasticity of individuals with math anxiety and explore whether these abnormalities are related to the clinical manifestations of math anxiety, thereby providing a more comprehensive and refined theoretical basis for future interventions against math anxiety.

(2) Optimization of intervention strategies

For math anxiety, future research should delve deeper into the optimization and sustainability of intervention strategies. Firstly, the range of intervention methods should be broadened. In addition to cognitive-behavioral therapy, psychoeducation, and relaxation training, considerations could be given to introducing psychodynamic therapy and acceptance and commitment therapy, enriching the diversity of interventions. Simultaneously, research should consider the combined use of different intervention methods to enhance the overall effect of the interventions. Secondly, the focus of research needs to extend from the short-term effects of interventions to long-term effects. Research should conduct long-term follow-ups to comprehensively assess the sustainability and stability of intervention strategies, ensuring their long-term application. Lastly, future research should also deeply understand the mechanisms of intervention actions, clarifying the precise pathways of their effects, and further optimizing the targeting and selection of intervention methods. Through these in-depth studies, it is expected to find more precise and effective strategies, providing continuous and comprehensive support for individuals troubled by math anxiety.

#### 4.8.2 Multidimensional exploration of cognitive impairments

(1) Improvement of diagnostic and assessment tools

Future research on cognitive impairments should focus on the multidimensional improvement and innovation of diagnostic and assessment tools. Firstly, research should focus on developing more accurate and sensitive diagnostic assessment tools. This involves not only the development of new psychological measurement tools but also the revision and optimization of existing tools to assess individuals’ cognitive impairments more comprehensively and accurately. Secondly, more advanced neuroimaging technologies should be utilized to deeply explore and assess cognitive impairments from a neurobiological perspective, aiming to reveal their deeper neural mechanisms. Lastly, by introducing machine learning and artificial intelligence technologies, significant information for diagnosing and assessing cognitive impairments can be identified in big data. By building and training more accurate predictive models, the risk and severity of cognitive impairments can be identified and assessed more effectively. The comprehensive application of these methods and technologies will help improve the accuracy and sensitivity of the diagnosis and assessment of cognitive impairments.

(2) Personalized intervention plans

The direction of future research should focus on the multidimensional exploration of cognitive impairments, especially in formulating personalized intervention plans. For different types and degrees of cognitive impairments, research should design more specific and targeted intervention strategies. For example, interventions for mild cognitive impairments can adopt more gentle and heuristic methods, while severe cognitive impairments require more intensive and systematic strategies. Additionally, the formulation of intervention plans should also fully consider individual differences, such as age, gender, educational background, and psychological characteristics. Thus, by integrating various factors, intervention plans can be more refined and personalized, thereby effectively improving the effectiveness and efficiency of interventions, promoting the improvement and recovery of individuals with cognitive impairments.

#### 4.8.3 Interaction research between math anxiety and cognitive impairments

(1) Exploration of mutual influence mechanisms

An essential direction for future research is to delve deeply into the interaction mechanisms between math anxiety and cognitive impairments. Firstly, research should reveal more precisely how math anxiety affects individuals’ performance in cognitive processes such as working memory, attention allocation, and information processing speed, and how this indirectly leads to the emergence and exacerbation of cognitive impairments. Simultaneously, attention should be given to how cognitive impairments become a source of math anxiety, considering how they enhance individuals’ math anxiety by causing learning difficulties and declining academic performance.

From a neurobiological perspective, future research could explore the common neural foundations of math anxiety and cognitive impairments. For instance, by utilizing brain imaging technologies, the similarities and differences in brain activity and connection patterns between math anxiety and cognitive impairments could be revealed. Also, considerations should be given to exploring their mutual influences on behavioral performances and learning strategies from psychological and educational perspectives. By integrating research methods and perspectives from different disciplines, a more comprehensive understanding of the interactions between math anxiety and cognitive impairments is expected, providing theoretical support for alleviating math anxiety and improving cognitive impairments.

(2) Comprehensive intervention strategies

Regarding the interactive relationship between math anxiety and cognitive impairments, the direction of future research should focus on developing comprehensive intervention strategies to address these two issues bidirectionally. Firstly, for math anxiety, psychological intervention strategies such as cognitive-behavioral therapy, psychoeducation, and relaxation training can be adopted to help individuals stabilize emotions and reduce anxiety levels. Simultaneously, to address cognitive impairments, educational interventions like metacognitive strategy training, problem-solving strategy training, and learning strategy guidance will be effective. A more innovative research direction might involve combining the aforementioned psychological and educational interventions to form a comprehensive intervention model. For example, integrating specific training for cognitive impairments into psychological interventions, or incorporating psychological support for math anxiety into educational interventions. Such comprehensive strategies can not only achieve comprehensive improvements in math anxiety and cognitive impairments but also contribute to providing a more systematic, in-depth understanding and intervention methods for these two major issues.

#### 4.8.4 Interdisciplinary collaborative research

In today’s complex and ever-changing academic research field, interdisciplinary collaborative research has become a trend and necessity, especially on cross-disciplinary issues like math anxiety and cognitive impairments, where experts and scholars from various disciplines need to join hands for exploration and research.

(1) Interdisciplinary integration

Future research on math anxiety should emphasize interdisciplinary integration and collaboration. Math anxiety is not merely a problem in the field of psychology; it is closely related to brain structure and function, educational methods, and individual psychology and emotional responses. Neuroscientists can explore how math anxiety affects brain activity using advanced brain imaging technologies, providing a scientific basis for formulating corresponding intervention measures. Educators can delve deeply into educational methods and strategies, exploring how to reduce the incidence of math anxiety through educational interventions and applying research findings to educational practice. The contribution of psychologists lies in deeply analyzing the psychological mechanisms of math anxiety, studying its association with other psychological problems, and looking for possibilities of comprehensive interventions. This interdisciplinary collaboration helps us understand the causes of math anxiety from different dimensions and levels comprehensively and find more effective intervention methods.

(2) International collaboration

With the acceleration of globalization, research on math anxiety and cognitive impairments also shows an increasingly international trend. Faced with this global issue, the importance of interdisciplinary and international collaboration becomes more prominent. International collaboration not only promotes resource sharing, improving research efficiency and quality, but also broadens research perspectives through the exchange and collision of different cultures and educational backgrounds, leading to the exploration of new research directions and methods. More importantly, international collaboration can promote the global dissemination and application of research findings, providing global strategies for solving problems of math anxiety and cognitive impairments. In conclusion, through deepening interdisciplinary integration and international collaboration, we can expect to promote the progress of research on math anxiety and cognitive impairments more quickly and effectively, providing more scientific and practical theoretical support and strategic suggestions for related educational practices.

Through in-depth research in the above directions, we can expect richer and more profound research findings in the fields of math anxiety and cognitive impairments, providing more scientific and effective theoretical support and strategic suggestions for educational practices.

## 5 Conclusion

### 5.1 Main findings

This review systematically analyzes a wealth of related literature, aiming to deeply explore the core roles of math anxiety and cognitive impairments in mathematical cognition processes and their interconnections. In terms of cognitive impairments, the study reveals two primary forms of impediments: developmental calculation disorders and acquired calculation disorders. The former typically manifests as inherent calculation deficiencies without apparent neurophysiological damage, while the latter usually results from some form of brain injury or disease causing the loss or decline of calculation abilities. However, appropriate training and intervention strategies may help improve or alleviate the symptoms of these impediments.

Regarding math anxiety, this phenomenon plays a crucial role in mathematical cognitive processes. Math anxiety not only affects the representation of numbers and calculations but also interferes with the resolution of arithmetic problems and spatial processing abilities. Individuals suffering from math anxiety may find mathematical tasks more challenging, leading to increased psychological stress and further decline in mathematical performance.

By integrating these findings, this article helps comprehensively understand how math anxiety and cognitive impairments mutually influence mathematical cognition at multiple levels. This understanding not only deepens our exploration of the psychological and neural mechanisms of mathematical cognition but also provides theoretical support for formulating precise and effective educational intervention strategies for various types of mathematical difficulties. Future research should further explore how to help individuals affected by math anxiety and cognitive impairments more effectively through integrated intervention methods, promoting their mathematical learning and self-development.

### 5.2 Practical applications

The profound findings of this study offer valuable insights into the educational field, especially concerning resolving students’ math anxiety and cognitive impairment issues. Firstly, for students with cognitive impairments, educators can design targeted and personalized teaching strategies and intervention measures based on individual differences. For instance, creating a more supportive learning environment and providing abundant practical opportunities to enhance their mathematical cognitive abilities, while stimulating students’ learning enthusiasm and confidence through cooperative learning and group discussions. Secondly, to alleviate students’ math anxiety, educators should strengthen care and support, helping students gradually overcome anxiety through more guiding and encouraging methods. Introducing lively and interesting teaching methods, such as games and stories, is also an effective way to improve students’ learning interest and participation. Additionally, educators can flexibly adjust teaching strategies to meet the specific needs of different students. For example, integrating more practical applications and case analyses into teaching, allowing students to feel the practicality of mathematical learning, enhancing learning enthusiasm and motivation. Simultaneously, through regular feedback and evaluation, educators can timely grasp students’ learning conditions, make corresponding teaching adjustments, and meet students’ learning needs more precisely.

In summary, this study provides educators with theoretical support and strategic guidance for practically addressing students’ math anxiety and cognitive impairments. Educators should fully utilize these research findings, scientifically and reasonably design and implement teaching activities, aiming to improve students’ mathematical cognitive abilities and learning outcomes, helping them overcome difficulties and challenges in the learning process.

## Author contributions

HY: Conceptualization, Writing – original draft, Writing – review & editing.
